# Mild Hypothermia Attenuates Hepatic Ischemia–Reperfusion Injury through Regulating the JAK2/STAT3-CPT1a-Dependent Fatty Acid *β*-Oxidation

**DOI:** 10.1155/2020/5849794

**Published:** 2020-03-20

**Authors:** Wei Wang, Xiaoyan Hu, Zhiping Xia, Zhongzhong Liu, Zibiao Zhong, Zhongshan Lu, Anxiong Liu, Shaojun Ye, Qin Cao, Yanfeng Wang, Fan Zhu, Qifa Ye

**Affiliations:** ^1^Zhongnan Hospital of Wuhan University, Institute of Hepatobiliary Diseases of Wuhan University, Transplant Center of Wuhan University, Hubei Key Laboratory of Medical Technology on Transplantation, Engineering Research Center of Natural Polymer-based Medical Materials in Hubei Province, Wuhan, China 430071; ^2^State Key Laboratory of Virology, Department of Medical Microbiology, School of Medicine, Wuhan University, Wuhan, China; ^3^The Third Xiangya Hospital of Central South University, Research Center of National Health Ministry on Transplantation Medicine Engineering and Technology, Changsha, China 410013

## Abstract

Hepatic ischemia–reperfusion (IR) injury is a clinical issue that can result in poor outcome and lacks effective therapies at present. Mild hypothermia (32–35°C) is a physiotherapy that has been reported to significantly alleviate IR injury, while its protective effects are attributed to multiple mechanisms, one of which may be the regulation of fatty acid *β*-oxidation (FAO). The aim of the present study was to investigate the role and underlying mechanisms of FAO in the protective effects of mild hypothermia. We used male mice to establish the experimental models as previously described. In brief, before exposure to in situ ischemia for 1 h and reperfusion for 6 h, mice received pretreatment with mild hypothermia for 2 h and etomoxir (inhibitor of FAO) or leptin (activator of FAO) for 1 h, respectively. Then, tissue and blood samples were collected to evaluate the liver injury, oxidative stress, and changes in hepatic FAO. We found that mild hypothermia significantly reduced the hepatic enzyme levels and the score of hepatic pathological injury, hepatocyte apoptosis, oxidative stress, and mitochondrial injury. In addition, the expression of the rate-limiting enzyme (CPT1a) of hepatic FAO was downregulated almost twofold by IR, while this inhibition could be significantly reversed by mild hypothermia. Experiments with leptin and etomoxir confirmed that activation of FAO could also reduce the hepatic enzyme levels and the score of hepatic pathological injury, hepatocyte apoptosis, oxidative stress, and mitochondrial injury induced by IR, which had the similar effects to mild hypothermia, while inhibition of FAO had negative effects. Furthermore, mild hypothermia and leptin could promote the phosphorylation of JAK2/STAT3 and upregulate the ratio of BCL-2/BAX to suppress hepatocyte apoptosis. Thus, we concluded that FAO played an important role in hepatic IR injury and mild hypothermia attenuated hepatic IR injury mainly via the regulation of JAK2/STAT3-CPT1a-dependent FAO.

## 1. Introduction

Hepatic ischemia–reperfusion (IR) injury is a serious clinical problem that can hardly be avoided in certain kinds of surgeries, such as liver transplantation and resection, as well as hemorrhagic shock [1, 2]. The paradigm of hepatic IR is based on two apparently separate phases, involving the ischemic phase and the reperfusion phase. The ischemic phase mainly causes cellular metabolic disturbances that result from glycogen consumption, lack of oxygen supply, and ATP depletion, while the reperfusion phase results not only in metabolic disturbances but also in a profound inflammatory immune response that involves both direct and indirect cytotoxic mechanisms [1]. The complicated IR injury is one of the main reasons for early graft failure and the increased risk of organ rejection and liver dysfunction [3]. Unfortunately, currently, the promising interventions for the treatment of hepatic IR injury are limited in number [4–6]. Thus, more effective strategies that can reduce hepatic IR injury and improve graft viability are urgently needed.

Mild hypothermia (32–35°C) [7] is a clinical therapy that is widely used in acute brain injury [8] and cardiac arrest [9], and there has been growing interest in the protective effects of mild hypothermia on IR injury in recent years. Our previous studies confirmed that mild hypothermia could significantly attenuate organ IR injury, and the protective effects are thought to be the result of interactions of multiple factors [10–15]. Based on these preliminary studies, we performed a proteomic analysis and found that the protective mechanisms of mild hypothermia were extremely complex. Therefore, it is of great importance to further study the protective mechanisms of mild hypothermia. This will not only significantly expand our knowledge on mild hypothermia but also greatly facilitate its clinical application.

As is well known, hypothermia could lead to a significant reduction in body metabolism; cellular oxygen and glucose requirements decrease by an average of 5–8% for every degree of decrease in temperature [7, 16, 17]. However, the effects of the decrease in temperature on hepatic lipid metabolism are complex and interconnected [18–21]. Cold exposure increases hepatic triglyceride (TG) concentrations but reduces hepatic lipogenic gene expression [18]. Hepatic expression of genes encoding proteins involved in cholesterol synthesis and uptake and classical bile acid (BA) synthesis is significantly increased upon cold exposure [18, 21]. After cold exposure, hepatic BA concentrations and fecal BA excretion are increased, while very low-density lipoprotein- (VLDL-) TG secretion is reduced [18, 21]. Thus, it is very important to clarify whether the complex effects of hypothermia on hepatic lipid metabolism are involved in hepatic IR injury.

The liver is a key regulatory organ in lipid metabolism; it can take up as well as oxidize fatty acids; it can synthesize, store, and secrete TG in VLDL particles; and it is the main metabolic orsgan that controls whole-body cholesterol and BA metabolism [18, 22]. More importantly, mitochondrial fatty acid *β*-oxidation (FAO) in the liver is the primary pathway for the oxidation of fatty acids and also the key metabolic pathway for energy homeostasis [23, 24]. Furthermore, the liver can convert the products of FAO into ketone bodies, which is an additional energy source in all tissues, including the brain [23]. A large number of enzymes are necessary for FAO, and deficiency in almost any of the enzymes involved in this process, especially the rate-limiting enzyme carnitine palmitoyltransferase 1 (CPT1), will result in lipid metabolic disturbances [23, 25, 26]. Metabolic disturbances of the lipid profile can induce inflammation, oxidative stress, and apoptosis by regulating relevant signaling transduction pathways, which can in turn exacerbate lipid metabolic reprogramming [27, 28]. Zhang et al. showed that the pathogenesis of hepatic IR injury was marked primarily by lipid metabolic reprogramming that leads to a secondary effect on inflammation, highlighting the importance of lipid metabolism in the pathogenesis of IR injury [29]. In view of these findings, fatty acid metabolism is currently attracting considerable interest in hepatic IR injury [30], and the role of FAO in IR injury is an important research topic.

Both mild hypothermia and IR injury have significant effects on lipid metabolism. However, the effects of changes in fatty acid metabolism under mild hypothermia on IR injury have not been addressed. Hence, the concrete effects of hepatic FAO under mild hypothermia on liver IR injury in male mice and the exact signaling pathway associated with these processes will be described in the following sections.

## 2. Materials and Methods

### 2.1. Animal Studies

**Figure 1:**
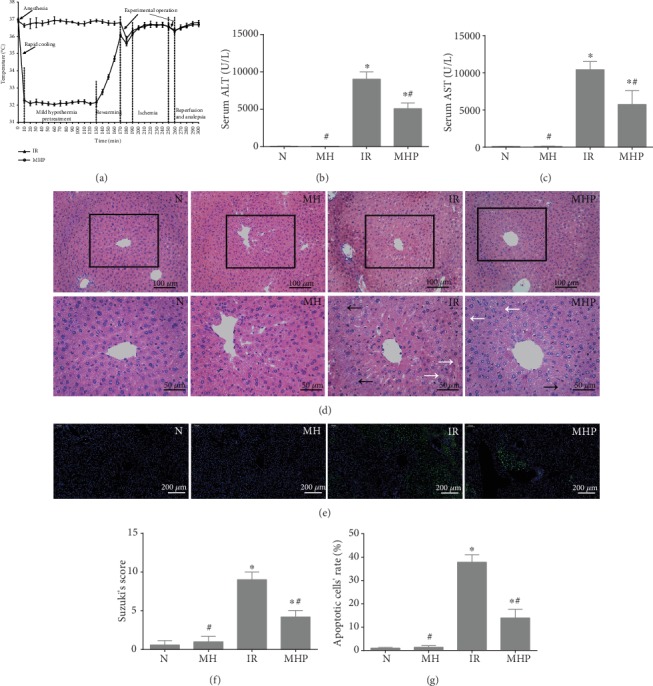
Mild hypothermia pretreatment attenuates hepatic IR injury. (a) Animal temperature changes throughout the experiment. (b) Serum ALT levels. (c) Serum AST levels. (d) Representative hematoxylin and eosin (HE) staining of liver tissues, the white “→” refers to sinusoidal congestion and the black “→” refers to necrosis. Original magnification, 200x and 400x. (e) Representative images of TUNEL staining, green fluorescence represents the TUNEL-positive cells. Original magnification, 100x. (f) Suzuki's histological score of liver tissue. (g) Quantitative analysis of apoptotic liver cells. *n* = 5 per group; data are expressed as mean ± SD; ^∗^*P* < 0.05 versus N group, ^#^*P* < 0.05 versus IR group; N: normal group; MH: mild hypothermia pretreatment group; IR: IR group; MHP: mild hypothermia pretreatment+IR group.

The experiments were performed with 6- to 8-week-old C57BL/6J male mice purchased from Wuhan Wan Qian Jia Xing Bio-Technology Co., Ltd. (Hubei, China). Mice were housed in the Animal Center of Zhongnan Hospital of Wuhan University and kept in a temperature-controlled environment (20-25°C) with a 12/12 h light/dark cycle with free access to food and water. The study was approved by the Ethical Committee of Wuhan University. All animal experiments were carried out in accordance with the Experimental Animal Management Ordinance (National Science and Technology Committee of China) and the Guide for the Care and Use of Laboratory Animals (National Institutes of Health (NIH), Bethesda, MD, USA). Animals fasted overnight while with access to water before the experiments. Mice were randomly divided into six groups (*n* = 5 for each group): (1) normal group (N), with mice only suffering a midline incision to expose the liver; (2) mild hypothermia pretreatment group (MH), with mice only receiving pretreatment with mild hypothermia; (3) IR group (IR), with mice exposed to in situ ischemia for 1 h and reperfusion for 6 h; (4) mild hypothermia pretreatment+IR group (MHP), with mice receiving pretreatment with mild hypothermia for 2 h and then exposure to IR; (5) etomoxir+IR group (EIR), with mice receiving pretreatment with etomoxir for 1 h and then exposure to IR; and (6) leptin+IR group (LIR), with mice receiving pretreatment with leptin for 1 h and then exposure to IR. The animal experiments were similar to those described previously [13]. Briefly, animals were anesthetized with sodium pentobarbital via i.p. injection (40 mg/kg body mass); (+)-etomoxir sodium salt hydrate (5 mg/kg, Sigma-Aldrich, E1905, USA) and Recombinant Mouse Leptin (5 mg/kg, Protein Specialists, cyt-351, USA), dissolved in saline, were administered i.p. 1 h before in situ warm ischemia. For mild hypothermia pretreatment, the animal core temperature was rapidly cooling to 32.0 ± 0.25°C with an ice blanket and kept for 2 h with a heating panel and an ice blanket at room temperature (20-25°C) and then rewarmed to 36.2 ± 0.2°C. Subsequently, a midline incision was made to expose the liver and free the perihepatic ligament; then, the branches of the portal vein and the hepatic artery that supply the left lateral and median lobes of the liver were occluded with an atraumatic Glover bulldog clamp for 1 h. Finally, the clamp was removed to initiate hepatic reperfusion and the abdominal midline incision was sutured. The whole experiment was conducted at room temperature (20-25°C), and the rectal temperature was monitored throughout the experiment (Figure 1(a)). After 6 h of reperfusion, mice were reanesthetized and sacrificed to collect livers and blood samples; 5 ml cold heparinized Ringer per animal was used via the abdominal aorta to flush the blood from the liver.

### 2.2. Biochemical Analysis

Blood was drawn from the postcava and centrifuged at 3500 rpm for 10 min. Serum was collected and stored at −80°C. Hepatocellular injury was determined by serum level of alanine aminotransferase (ALT) and aspartate aminotransferase (AST) by automatic analysis in the Zhongnan Hospital of Wuhan University.

### 2.3. Histopathology and TUNEL Staining

Ischemic lobes were harvested and fixed in 4% formalin. Samples were embedded in paraffin as previously described [13]. All paraffin sections for histological observation were stained with hematoxylin and eosin (H&E), and tissue sections of IR injury were graded blindly by Suzuki's criteria [31]. Histological changes were graded from 0 to 4 based on the degree of cellular vacuolization, hepatic sinusoid congestion, and hepatocyte necrosis.

Apoptosis was assayed by Terminal deoxynucleotidyl transferase-mediated dUTP nick end labeling (TUNEL) staining following the manufacturer's instructions. The total hepatocytes and TUNEL-positive cells were detected in three randomly chosen views (100x) for each liver section using a fluorescence microscope. The rate of apoptosis (number of TUNEL − positive cells/total number of hepatocytes × 100 %) in each view was calculated with Image-Pro Plus 6.0 (Media Cybernetics, Rockville, MD, USA).

### 2.4. Electron Microscopy

Fresh ischemic livers were fixed in 2.5% glutaraldehyde and washed with PBS for 15 min three times. Samples were fixed in 1% osmic acid for 1–2 h followed by washing with PBS for 15 min three times again. Next, samples were dehydrated with a graded series of ethanol solutions (50%, 70%, 80%, 90%, and 95%) for 15 min at each concentration and then treated with ethanol and acetone for 20 min each. Next, samples were treated with a mixture of embedding agent and acetone (*V* : *V* = 1 : 1) for 1 h and (*V* : *V* = 3 : 1) for 3 h, then treated with embedding agent overnight, and heated at 70°C overnight. Finally, samples were sliced to 70–90 nm sections in a Reichert ultrathin slicer and then stained with lead citrate solution and uranyl acetate 50% ethanol-saturated solution for 15 min for observation.

### 2.5. Western Blot Analysis

Western blot was performed using whole lysates extracted from livers as previously described [13]; phosphatase inhibitors were purchased from Roche. The primary antibodies used in these experiments were the following: rabbit anti-JAK2 (1 : 750, Proteintech, Manchester, UK), rabbit anti-p-JAK2 (phosphorylated JAK2 at Tyr1007/1008) (1 : 1000, Cell Signaling, Danvers, MA, USA), rabbit anti-STAT3 (1 : 750, Proteintech, Manchester, UK), mouse anti-p-STAT3 (phosphorylated STAT3 at Tyr705) (1 : 1000, Cell Signaling, Danvers, MA, USA), rabbit anti-ACSL1 (1 : 750, Proteintech, Manchester, UK), rabbit anti-CPT1a (1 : 1000, Proteintech, Manchester, UK), rabbit anti-ACADVL (1 : 750, Proteintech, Manchester, UK), rabbit anti-HADHA (1 : 1000, Proteintech, Manchester, UK), rabbit anti-HMGCS1 (1 : 750, Proteintech, Manchester, UK), mouse anti-BCL-2 (1 : 1000, Cell Signaling, Danvers, MA, USA), rabbit anti-BAX (1 : 750, Proteintech, Manchester, UK), rabbit anti-PFKM (1 : 1000, Proteintech, Manchester, UK), rabbit anti-IDH2 (1 : 1000, Proteintech, Manchester, UK), and rabbit anti-CS (1 : 1000, Proteintech, Manchester, UK). Blots were incubated and visualized with enhanced chemiluminescence (ECL) reagent (Proteintech, Manchester, UK). The protein expression levels were normalized to *β*-actin (1 : 3000, mouse anti-*β*-actin antibody, Proteintech, Manchester, UK) and GAPDH (1 : 2000, rabbit anti-GAPDH antibody, Proteintech, Manchester, UK). The protein expression was quantified by densitometric analysis using the Image J software.

### 2.6. CPT1, ATP, ADP/ATP, NAD^+^/NADH, Acetyl CoA, and Malonyl CoA Analysis

For the evaluation of mitochondrial function and FAO, frozen liver tissue was homogenized with specified buffer that was included in the ELISA kits specific for CPT1 (Nanjing Jiancheng Bioengineering Institute, Nanjing, China), adenosine triphosphate (ATP) (Nanjing Jiancheng Bioengineering Institute, Nanjing, China), ADP/ATP Ratio Assay Kit (Abnova Corporation, Taiwan, China), NAD^+^/NADH Assay Kit (Abnova Corporation, Taiwan, China), acetyl coenzyme A (Acetyl CoA) (Elabscience Biotechnology Co., Ltd., Wuhan, China), and malonyl coenzyme A (Malonyl CoA) (Biorbyt Ltd., Cambridge, UK); detections were performed following the manufacturers' instructions. The results were measured as ng/ml, *μ*mol/gprot, *μ*g/g, and *μ*g/g.

### 2.7. Superoxide Dismutase (SOD), Malondialdehyde (MDA), ROS, and 4-HNE Analysis

For the evaluation of oxidative stress, frozen liver tissue was homogenized with specified buffer that was included in the colorimetric assay kits specific for superoxide dismutase (SOD), malondialdehyde (MDA), reactive oxygen species (ROS) (Nanjing Jiancheng Bioengineering Institute, Nanjing, China), and 4-hydroxynonenal (4-HNE) (Elabscience Biotechnology Co., Ltd., Wuhan, China), which were detected following the manufacturers' instructions. The results were measured as U/mgprot, nmol/mgprot, FI/mg, and ng/g.

### 2.8. Statistical Analysis

Data were analyzed using SPSS 19.0 statistical software for Windows (SPSS Inc., Chicago, IL, USA). All results are presented as mean ± SD. Differences between experimental groups were analyzed by one-way analysis of variance (ANOVA); *P* < 0.05 was considered statistically significant.

## 3. Results

### 3.1. Mild Hypothermia Attenuates Hepatic IR Injury in Mice

We monitored the rectal temperature throughout the experiment to ensure the stability of the experimental model; data is shown in Figure 1(a). To confirm the protective effects of mild hypothermia against IR injury, hepatic function, hepatic architecture distortion, and hepatocyte apoptosis rates were measured. As shown in Figures 1(b) and 1(c), compared with the normal group, the levels of ALT and AST were significantly increased after IR, i.e., from 51.5 ± 23 U/L and 87.4 ± 39.2 U/L to 9029.8 ± 981.8 U/L and 10434.8 ± 1094.8 U/L, respectively; this increase could be greatly alleviated by mild hypothermia (*P* < 0.05), as ALT and AST levels decreased to 5071.2 ± 776 U/L and 5735.2 ± 1886.3 U/L. Meanwhile, hepatic histopathology changes were assessed. As shown in Figures 1(d) and 1(f), IR injury resulted in serious cytoplasmic vacuolization of hepatocytes, severe necrotic areas, and moderate vascular and sinusoidal congestion, while only mild cytoplasmic vacuolization and necrosis of hepatocytes and almost no vascular and sinusoidal congestion could be observed after pretreatment with mild hypothermia. The score of hepatic pathological injury increased from 0.6 ± 0.5 to 9 ± 1 after IR injury and decreased to 4.2 ± 0.8 after pretreatment with mild hypothermia (*P* < 0.05). Similarly, as shown in Figures 1(e) and 1(g), mild hypothermia could also reduce the apoptosis rate upon IR injury; it decreased from 37.82% ± 3.18% to 13.94% ± 3.67% (*P* < 0.05). In addition, the hepatic functional and morphological changes and cell apoptosis did not exhibit a significant difference between the normal group and the mild hypothermia pretreatment group (*P* > 0.05). These results further confirm our previous research findings that mild hypothermia could attenuate IR injury.

### 3.2. Pretreatment with Mild Hypothermia Ameliorates Mitochondrial Injury and Oxidative Stress after Hepatic IR Injury

**Figure 2:**
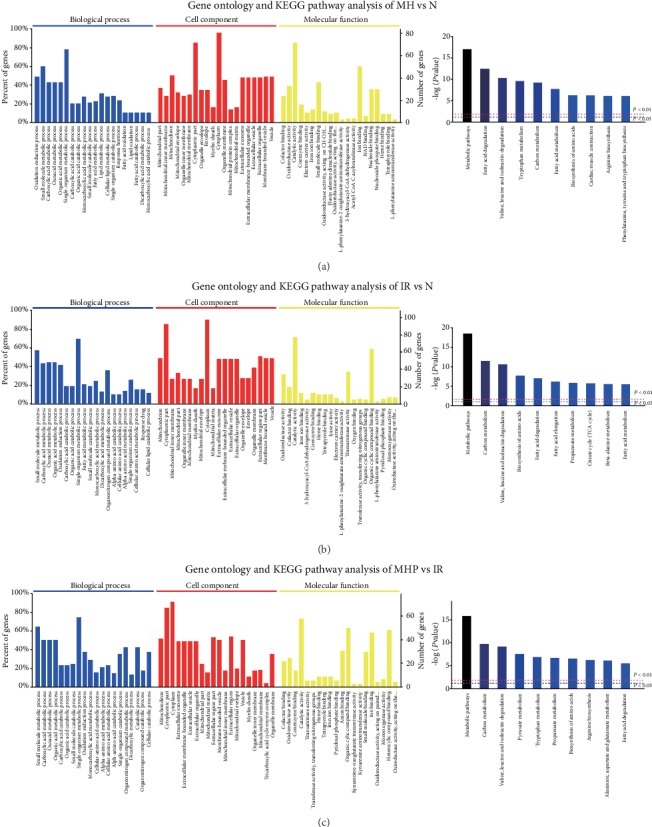
Gene ontology (GO) and Kyoto Encyclopedia of Genes and Genomes (KEGG) pathway analysis of differentially expressed genes. (a–c) GO annotation via the top 20 enrichment scores in the BP, CC, and MF domains and KEGG pathway analysis of the top 10 enrichment pathways of the MH vs. N groups (a), the IR vs. N groups (b), and the MHP vs. IR groups (c). *P* < 0.05 means significant difference; *P* < 0.01 means highly significant difference.

**Figure 3:**
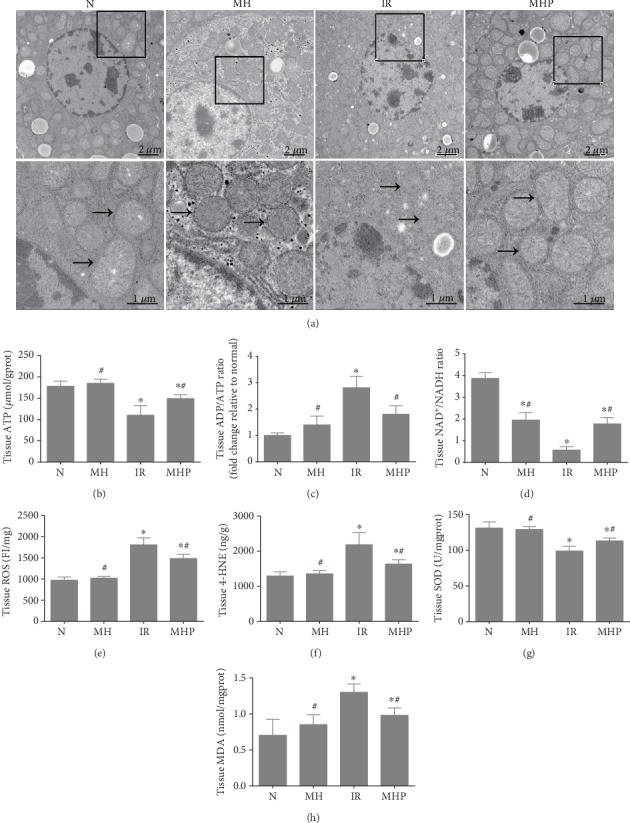
Mild hypothermia pretreatment alleviates mitochondrial injury and oxidative stress induced by hepatic IR. (a) Representative transmission electron microscope images of liver tissues, “→” refers to mitochondria. Original magnification, 1700x and 5000x. (b–d) Mitochondrial function was evaluated by detecting (b) ATP levels, (c) ADP/ATP Ratio, and (d) NAD^+^/NADH Ratio in liver tissue. (e–h) The levels of oxidative stress were estimated by measuring the levels of (e) ROS, (f) 4-HNE, (g) SOD, and (h) MDA. *n* = 5 per group; data are expressed as mean ± SD; ^∗^*P* < 0.05 versus N group, ^#^*P* < 0.05 versus IR group.

Based on the aforementioned results and our preliminary studies, we performed a proteomic analysis and found that metabolism, especially lipid metabolism, was markedly changed in these animal models (see Figure 2); this suggests that lipid metabolism may play an important role in mild hypothermia which attenuates hepatic IR injury. Since mitochondria are the main intracellular site of metabolism, transmission electron microscopy was used to observe the mitochondrial morphological changes; ATP levels, ADP/ATP Ratio, and NAD^+^/NADH in liver tissue were measured to evaluate the mitochondrial functional changes. As shown in Figure 3(a), the mitochondria were seriously injured by IR; we observed severe mitochondrial swelling, and most mitochondria were blurry or invisible; however, the mitochondrial morphology was much better after mild hypothermia pretreatment, as only moderate swelling could be seen, and most mitochondria were clear. Furthermore, the mitochondrial function were evaluated, as shown in Figures 3(b)–3(d), the ATP levels and NAD^+^/NADH were significantly decreased, and ADP/ATP Ratio was significantly increased after IR injury, while mild hypothermia could improve these changes in the liver. These results were consistent with the mitochondrial morphological changes (*P* < 0.05). Moreover, mitochondrial injury induced by IR resulted in increased ROS, 4-HNE, and MDA levels and decreased SOD levels, which are biomarkers of lipid peroxidation and oxidative stress. Pretreatment with mild hypothermia could also significantly prevent these changes and thus ameliorates lipid peroxidation and reduces oxidative stress injury (*P* < 0.05) (see Figures 3(e)–3(h)). Likewise, the levels of mitochondrial injury, lipid peroxidation, and oxidative stress were not significantly different between the normal group and the mild hypothermia pretreatment group (*P* > 0.05). These results show that mild hypothermia could reduce IR-induced mitochondrial injury, lipid peroxidation, and oxidative stress.

### 3.3. Mild Hypothermia Regulates Fatty Acid Oxidation in Hepatic IR Injury

**Figure 4:**
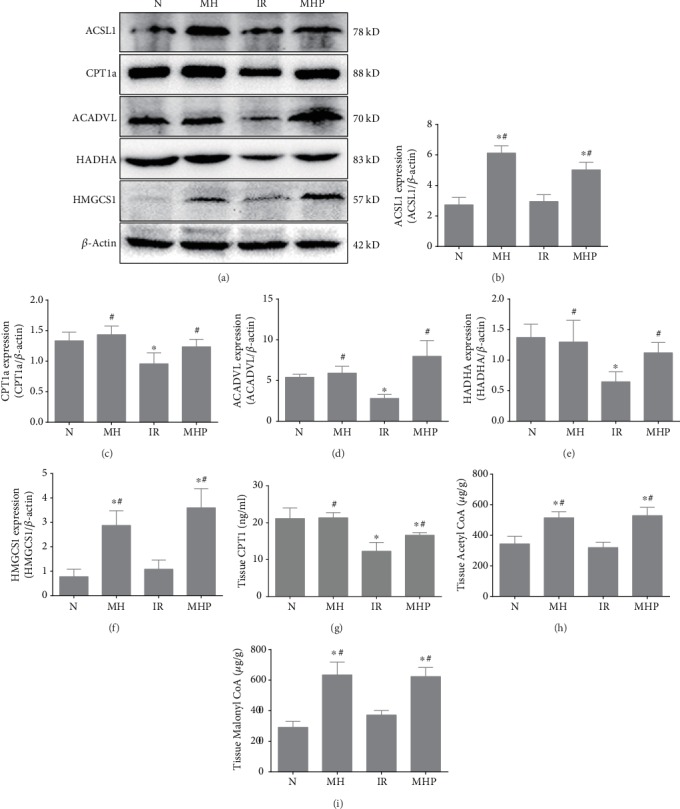
Mild hypothermia regulates FAO in hepatic IR injury. (a) Representative blots of proteases that are related to FAO. (b–f) Protein expression levels of (b) ACSL1, (c) CPT1a, (d) ACADVL, (e) HADHA, and (f) HMGCS1. The gray values were calculated, and protein expression levels were normalized to *β*-actin. (g) CPT1 levels in liver tissue were measured by ELISA. (h) Acetyl CoA levels in liver tissue. (i) Malonyl CoA levels in liver tissue. *n* = 5 per group; data are expressed as mean ± SD; ^∗^*P* < 0.05 versus N group, ^#^*P* < 0.05 versus IR group.

To test whether regulation of FAO was involved in mild hypothermia which attenuates hepatic IR injury, we evaluated the levels of proteases that are involved in the process of FAO and the associated products of FAO in liver tissue, such as ATP, Acetyl CoA, and Malonyl CoA. As shown in Figures 3(b) and 4, FAO was markedly inhibited by IR. Levels of CPT1a, the rate-limiting enzyme of hepatic FAO, which converts acyl-CoA into acylcarnitine, were downregulated nearly twofold; HADHA, which exhibits hydratase, long chain hydroxyacyl-CoA dehydrogenase, and thiolase activity and is critical for the *β*-oxidation cycle, was downregulated nearly twofold as well. Other proteases that participate in FAO were also obviously downregulated. The expression levels of these key enzymes could, however, be preserved by mild hypothermia (*P* < 0.05). Moreover, the production of ATP was also significantly decreased upon IR injury, whereas these changes could be reversed by pretreatment with mild hypothermia (*P* < 0.05). However, the production of Acetyl CoA and Malonyl CoA and the expression of HMGCS1, the rate-limiting enzyme of ketogenesis, did not exhibit significant differences between the normal group and the IR group (*P* > 0.05); they were, however, significantly different from the mild hypothermia pretreatment groups, with or without IR (*P* < 0.05). These results further highlight the notable effects of mild hypothermia on FAO. Thus, we conclude that mild hypothermia alleviates hepatic IR injury, primarily by regulating FAO.

### 3.4. Fatty Acid Oxidation Plays an Important Role in Hepatic IR Injury

**Figure 5:**
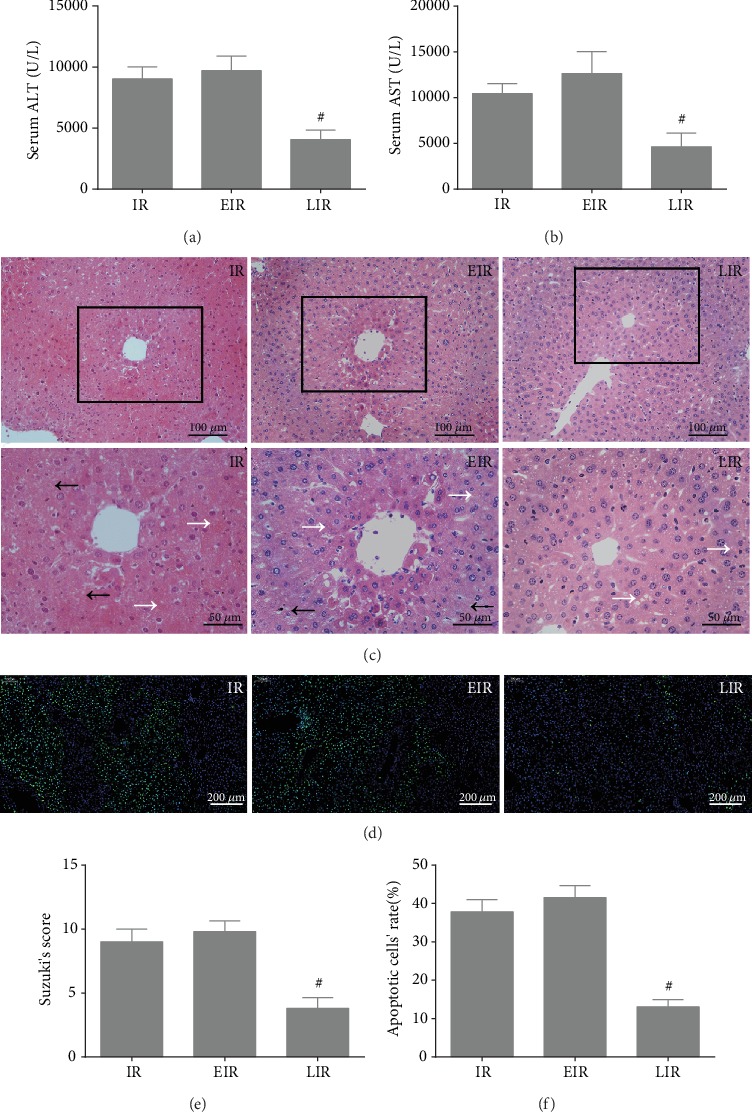
The effects of pharmacological regulation of FAO on hepatic IR injury. (a) Serum ALT levels. (b) Serum AST levels. (c) Representative images of hematoxylin and eosin- (HE-) stained liver tissues, the white “→” refers to sinusoidal congestion and the black “→” refers to necrosis. Original magnification, 200x and 400x. (d) Representative images of TUNEL staining, green fluorescence represents the TUNEL-positive cells. Original magnification, 100x. (e) Suzuki's histological score of liver tissue. (f) Quantitative analysis of apoptotic liver cells. *n* = 5 per group; data are expressed as mean ± SD; ^#^*P* < 0.05 versus IR group; EIR: etomoxir+IR group; LIR: leptin+IR group.

**Figure 6:**
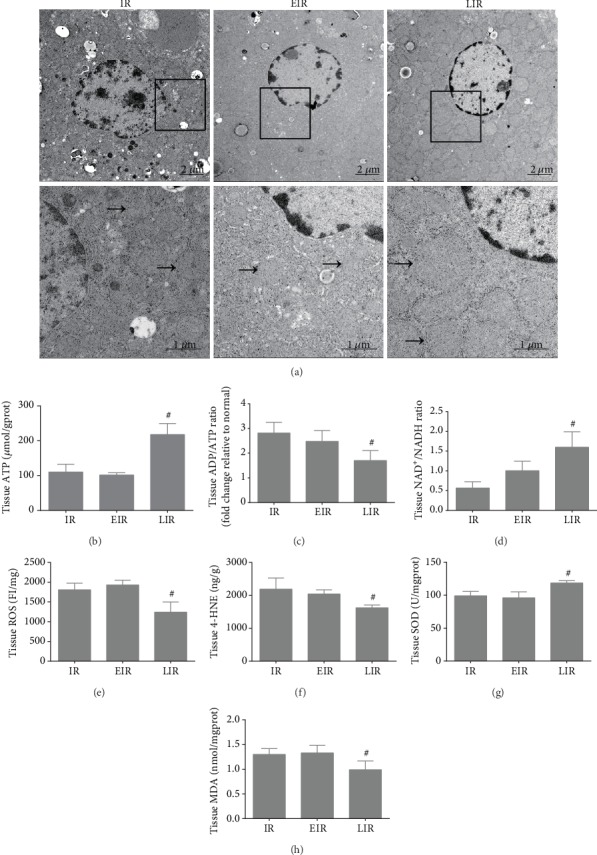
Regulation of FAO affects mitochondrial injury and oxidative stress induced by hepatic IR. (a) Representative transmission electron microscopy images of liver tissues, “→” refers to mitochondria. Original magnification, 1700x and 5000x. (b–d) The (b) ATP levels, (c) ADP/ATP Ratio, and (d) NAD^+^/NADH ratio in liver tissue were measured to evaluate mitochondrial function. (e–h) The levels of oxidative stress were estimated by measuring the levels of (e) ROS, (f) 4-HNE, (g) SOD, and (h) MDA. *n* = 5 per group; data are expressed as mean ± SD; ^#^*P* < 0.05 versus IR group.

**Figure 7:**
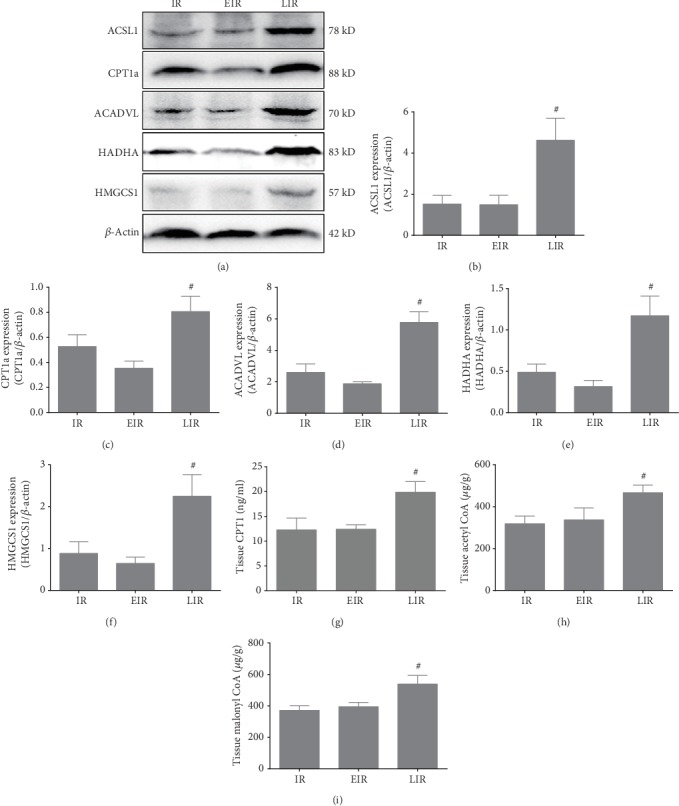
FAO plays an important role in hepatic IR injury. (a) Representative blots of proteases that are related to FAO. (b–f) Protein expression levels of (b) ACSL1, (c) CPT1a, (d) ACADVL, (e) HADHA, and (f) HMGCS1. The gray values were calculated, and protein expression levels were normalized to *β*-actin. (g) CPT1 levels in liver tissue were measured by ELISA. (h) Acetyl CoA levels in liver tissue. (i) Malonyl CoA levels in liver tissue. *n* = 5 per group; data are expressed as mean ± SD; ^#^*P* < 0.05 versus IR group.

After obtaining the above findings, pharmacological interventions of FAO were carried out to further illuminate the role of FAO in hepatic IR injury. Therefore, etomoxir, a specific inhibitor of FAO, and leptin, an FAO activator, were used in this experiment; their effects on CPT1a expression were shown in [Supplementary-material supplementary-material-1]. As shown in Figure 5, the use of etomoxir could worsen hepatic IR injury to some extent but it was not statistically significant; the levels of ALT and AST, the score of HE, and the rate of hepatocyte apoptosis did not exhibit significant difference with the IR group (*P* > 0.05). However, the application of leptin could significantly reduce the damage; levels of ALT and AST were reduced to 4065.6 ± 759.7 U/L and 4632.2 ± 1500 U/L, respectively, the score of hepatic pathological injury decreased to 3.8 ± 0.8, and the hepatocyte apoptosis rate was lowered to 13.06% ± 1.92% (*P* < 0.05). In addition, inhibition of FAO had negative effects but activation of FAO had positive effects on mitochondrial injury and oxidative stress, i.e., less-damaged mitochondrial morphology, decreased ROS, 4-HNE, MDA levels, and ADP/ATP Ratio, and increased SOD and ATP levels, and NAD^+^/NADH were observed after pretreatment with leptin (*P* < 0.05); these were consistent with the observed hepatic functional and morphological changes (see Figure 6). Furthermore, levels of the related proteases, Acetyl CoA, Malonyl CoA, and ATP were also changed accordingly, as the activation of FAO could upregulate the expression of these critical enzymes and the production of Acetyl CoA, Malonyl CoA, and ATP, and the effects are similar to those of mild hypothermia upon IR injury (*P* < 0.05), while inhibition of FAO had the opposite effects (see Figures 6(b) and 7). These results further highlight the important role of FAO in mild hypothermia-induced alleviation of hepatic IR injury.

In addition, glycolysis and TCA cycle were also involved in IR injury. Phosphofructokinase (PFKM), which is the key enzyme of glycolysis, and Citrate Synthase (CS) and Isocitrate Dehydrogenase 2 (IDH2), which are the key enzymes of TCA cycle, were downregulated after IR injury, while these changes could be preserved by mild hypothermia pretreatment and leptin. However, these changes were not as significant as those in FAO (see [Supplementary-material supplementary-material-1]).

### 3.5. Mild Hypothermia Ameliorates Liver IR Injury through Regulating the JAK2/STAT3 Pathway

**Figure 8:**
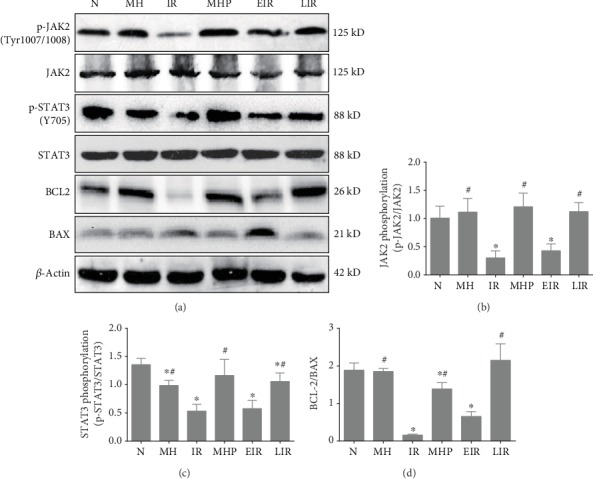
Mild hypothermia maintains the activation of the JAK2/STAT3 pathway after hepatic IR injury. (a) Representative blots of JAK2, STAT3, and important markers of apoptosis. (b) The phosphorylation levels of JAK2 at Tyr1007/1008. (c) The phosphorylation levels of STAT3 at Tyr705. (d) The ratio of BCL-2/BAX.

To further illustrate the predominant signaling pathway involved in these processes, we measured the activation of the JAK2/STAT3 pathway, which is upstream of CPT1 and downstream of leptin. As shown in Figure 8, the phosphorylation levels of both JAK2 and STAT3 were downregulated after IR injury. Similarly, inhibition of FAO inhibited protein phosphorylation, whereas activation of FAO and mild hypothermia could maintain the phosphorylation levels of JAK2 and STAT3 (*P* < 0.05). Moreover, the apoptosis-related proteins BCL2 and BAX showed significant changes; that is, the ratio of BCL2/BAX increased after activation of FAO and pretreatment with mild hypothermia, while it decreased after inhibition of FAO (*P* < 0.05). These results strongly suggest that activation of the JAK2/STAT3 pathway is closely associated with the protective effects of mild hypothermia against hepatic IR injury. These suggest that the JAK2/STAT3 pathway is of great importance in the protective effects of mild hypothermia against IR injury.

## 4. Discussion

Hepatic IR injury is a complex pathological process that can result in serious liver injury and severely threatens the short- and long-term prognosis of the patients who undergo liver surgery [3, 32, 33]. The limited number of currently available therapeutic strategies for IR injury urged us to explore more methods that can effectively prevent and attenuate IR injury. Recently, mild hypothermia attracted scholars' attention due to its profound protective effect and limited side effects; besides that, it is easy to reach and maintain and has been proven to be safe to patients [7 – 9, 16] and it also has been proven to have a positive effect on kidney transplantation from organ donors after declaration of death [34]. More importantly, our previous studies have preliminarily confirmed that mild hypothermia can significantly alleviate inflammation and apoptosis induced by IR injury [10 – 13]. In the present study, we used a well-established animal model of hepatic IR injury with or without mild hypothermia pretreatment. The results showed that mild hypothermia could significantly attenuate IR-induced hepatic functional and morphological injury, which were consistent with our previous studies. However, the underlying mechanisms by which mild hypothermia exerts protective effects still needs further research.

In order to elucidate the underlying mechanisms, we performed a proteomic analysis and found that metabolism exhibited significant changes upon IR injury and mild hypothermia pretreatment, of which, lipid metabolism, especially fatty acid oxidation, was of great significance. As is well known, the liver is one of the most important organs for FAO, and mitochondria are the main site where metabolism occurs. Therefore, we evaluated the mitochondrial morphological and functional changes and the hepatic FAO activity; results showed the IR-induced mitochondrial injury and FAO inhibition were largely reversed by mild hypothermia, thus demonstrating for the first time that FAO is one of the potential mechanisms by which mild hypothermia protects against IR injury. These results are consistent with previous studies, which concluded that metabolic disturbances during ischemia are a necessary precursor for reperfusion-induced inflammation and could also serve as a molecular target for therapeutic intervention [29].

FAO plays a pivotal role in energy homoeostasis, and the process is extremely complex. Briefly, after translocation across the plasma membrane, fatty acids are rapidly converted into acyl-CoA by acyl-CoA synthetases such as long-chain-fatty-acid-CoA ligase 1 (ACSL1) [23, 35, 36]. Then, acyl-CoA is converted to acylcarnitine by CPT1 at the mitochondrial outer membrane. CPT1 is the rate-limiting enzyme of FAO; it contains three isoforms, i.e., CPT1a (liver isoform), CPT1b (muscle isoform), and CPT1c (brain isoform) [37, 38]. Once acylcarnitine enters the mitochondria, it reconverted into CoA ester by carnitine palmitoyltransferase 2 (CPT2), and the CoA ester can finally enter the *β*-oxidation cycle [23]. The *β*-oxidation cycle is a process in which acyl-CoA is shortened, whereby the two carboxy-terminal carbon atoms are released as acetyl-CoA units for each cycle. In short, directly after import, acyl-CoA is metabolized first by an acyl-CoA dehydrogenase, such as very long-chain acyl-CoA dehydrogenase (ACADVL), and then by mitochondrial trifunctional protein (HADHA), which exerts hydratase, long-chain hydroxyacyl-CoA dehydrogenase, and thiolase activity [23]. The process of *β*-oxidation produces large amounts of acetyl-CoA, flavin adenine dinucleotide (FADH_2_), and nicotinamide adenine dinucleotide (NADH). Of the processes, acetyl-CoA can enter the citric acid cycle to produce ATP, it can be converted into ketones upon the catalysis of hydroxymethylglutaryl-CoA synthase (HMGCS1), and it can also be converted into malonyl-CoA. Moreover, FADH_2_ and NADH can be used to produce ATP [23]. The impaired mitochondrial function would not only result in severe disturbance of lipid metabolism but also increase the production of ROS, thus leading to serve lipid peroxidation and oxidative stress injury [24, 39]. In the present study, we show that IR injury can cause serious mitochondrial damage, downregulated expression of key enzymes in FAO, and also, a decreased rate of FAO. Furthermore, IR injury can also increase the production of ROS, which is one of the most common free radicals, and then lead to the increase in 4-HNE and MDA and decrease in SOD. However, these changes could be significantly reversed by pretreatment with mild hypothermia. These observations demonstrate that the protective effects of mild hypothermia on IR injury are mainly due to the preservation of mitochondrial FAO.

To further confirm the above results, *in vivo* pharmacological interventions of FAO were performed. Etomoxir, a small molecule developed for metabolic and cardiovascular disease that exhibits nanomolar potency toward CPT1 upon enzymatic conversion to the active inhibitor etomoxiryl-CoA, was used to block mitochondrial FAO [40]. At the same time, leptin, one of the adipokines that can regulate appetite and food intake, basal metabolism, vascular function, reproductive function, insulin secretion, inflammation, and immunity, was used to activate mitochondrial FAO [41–43]. The results of our study show that activation of FAO by leptin significantly alleviated hepatic IR injury, the effects were similar to those of mild hypothermia on IR injury. However, inhibition of FAO by etomoxir had negative effects on IR injury. These results demonstrate that the regulation of FAO plays an important role in IR injury and is a potential mechanism by which mild hypothermia attenuates hepatic IR injury.

Based on the above results, we investigated the main signaling pathway involved in these processes. Several pathways have been demonstrated to be associated with FAO, including the PPAR*α* pathway [24, 44], the AMPK pathway [45], and the JAK2/STAT3 pathway [46]. These pathways are also closely related to IR injury. The JAK2/STAT3 pathway is directly downstream of leptin, and JAK2/STAT3 can be phosphorylated after leptin binds to the leptin receptor (LEPR) [41]. Phosphorylation of JAK2/STAT3 leads to their dissociation from the receptor and the formation of active dimers, which translocate to the nucleus to regulate gene expression, thus regulating metabolism, reproduction, oxidative stress, apoptosis, and inflammation [47, 48]. As we know, apoptosis is one of the most important mechanisms of IR injury; thus, we evaluated the protein expression levels of BCL-2 and BAX in liver tissue to explore the role of mild hypothermia and FAO regulation on apoptosis upon IR injury. Our results show that the phosphorylation of JAK2/STAT3 and the ratio of BCL-2/BAX were decreased after IR injury, while mild hypothermia significantly prevented these changes. In addition, treatment with leptin also increased the phosphorylation of JAK2/STAT3 and the ratio of BCL-2/BAX, whereas etomoxir pretreatment did not cause any significant changes. These results suggest that the main signaling pathway involved in the protective mechanisms of mild hypothermia against IR injury is the JAK2/STAT3-CPT1a pathway.

Importantly, regarding the protective effects of leptin on hepatic IR injury, Lin et al. first found that endogenous leptin fluctuates in hepatic IR injury [49] and Carbone et al. suggested that leptin could reduce hepatic IR injury [50], but they both did not explore the underlying mechanisms. Our results are consistent with their findings and confirm the potential protective effects. In addition, our study showed that the phosphorylation levels of JAK2/STAT3 are downregulated after IR injury, while pretreatment with mild hypothermia and leptin can upregulate these and thus attenuate IR injury; these results confirmed that activating JAK2/STAT3 pathway can attenuate IR injury. However, our results are in dispute with some studies, which showed that the phosphorylation levels of JAK2/STAT3 are increased after IR injury [51–53]. We think it has something to do with the time of the models. The model of our study is *in vivo* hepatic ischemia for 1 h and reperfusion for 6 h, the time is relatively short, while the time of the models in the studies opposite to us are relative long, which often is more than 24 h. In addition, there are also many studies consistent with our results [54–56]; the time of their models is also relatively short, which is often less than 10 h. This means that the activation of JAK2/STAT3 may have different patterns at different time points after IR injury, and this need to be confirmed by our further research. All in all, regardless of the phosphorylation of JAK2/STAT3 after IR injury, one thing all the studies have in common is that activating the JAK2/STAT3 pathway can attenuate IR injury. Nevertheless, we wish to mention two limitations to our study. First, the mechanism by which JAK2/STAT3 affects CPT1 expression thus regulates FAO and reduces mitochondrial injury requires further study. Second, we only conducted *in vivo* intervention experiments, and we did not verify our findings by *in vitro* experiments or clinical trials.

## 5. Conclusion

**Figure 9:**
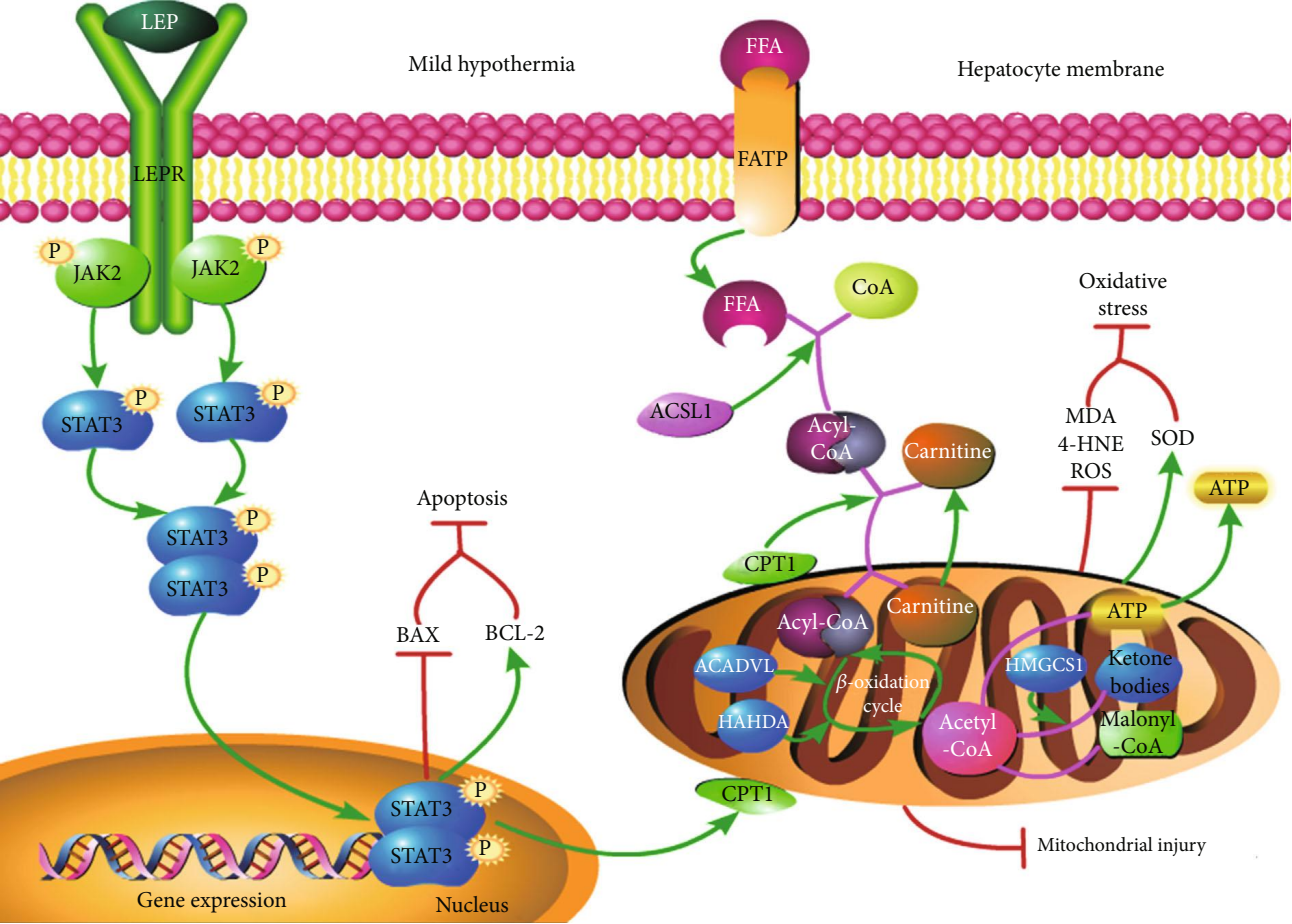
Schematic representation illustrating the potential mechanisms behind the protective effects of mild hypothermia against hepatic IR injury. Pretreatment with mild hypothermia promotes the homodimerization of leptin (LEP) and leptin receptor (LEPR), leading to the phosphorylation of JAK2/STAT3 and hence the activation of the signal transduction pathway. Activation of the JAK2/STAT3 pathway leads to the upregulation of the expression of CPT1, which is the rate-limiting enzyme of FAO and thereby accelerates hepatic FAO and ATP production. Sufficient ATP levels will alleviate IR-induced mitochondrial injury, inhibit the release of ROS, and reduce lipid peroxidation, thus attenuating oxidative stress. Furthermore, activation of the JAK2/STAT3 pathway promotes the expression of BCL-2 and inhibits the expression of BAX, thereby reducing hepatocyte apoptosis.

The major novel conclusions of the present study were the following. (1) Mild hypothermia effectively attenuates IR injury. (2) One of the mechanisms by which mild hypothermia exerts protective effects against IR injury is the preservation of mitochondrial FAO. (3) Pharmacological interventions of FAO have significant effects on IR injury; that is, activation of FAO can significantly attenuate IR injury, while inhibition of FAO has negative effects. (4) The JAK2/STAT3-CPT1a signaling pathway may play a vital role in these processes. A schematic representation of the underlying mechanisms is presented in Figure 9. Our results strongly support the notion that pretreatment with mild hypothermia is a feasible strategy to prevent hepatic IR injury, and we elucidated one of the possible mechanisms of the protective effects of mild hypothermia; these further improved our knowledge on mild hypothermia and provide a theoretical basis for further clinical application of mild hypothermia in liver surgery.

## Data Availability

The original experimental data used to support the findings of this study are available from the corresponding author (Qifa Ye, yqf_china@163.com) upon request.

## Supplementary Materials

Supplementary Materials Figure S1: the effects of Etomoxir and Leptin on CPT1a expression. (A, B) Representative blots of CPT1a after the use of Etomoxir and Leptin with different dose and time. (C, D) Protein expression levels of CPT1a after the use of Etomoxir and Leptin with different dose and time. The gray values were calculated, and protein expression levels were normalized to *β*-actin. *n* = 3 per group; data are expressed as mean ± SD; ^∗^*P* < 0.05 versus N group. Figure S2: the effects of mild hypothermia on glycolysis and TCA cycle. (A) Representative blots of PFKM, the key enzyme of glycolysis. (B) Representative blots of CS and IDH2, the key enzymes of TCA cycle. (C) Protein expression level of PFKM. (D) Protein expression levels of CS and IDH2. The gray values were calculated, and protein expression levels were normalized to GAPDH. *n* = 5 per group; data are expressed as mean ± SD; ^∗^*P* < 0.05 versus N group, ^#^*P* < 0.05 versus IR group.
