# Supplementary material for: Mild Hypothermia Attenuates Hepatic Ischemia–Reperfusion Injury through Regulating the JAK2/STAT3-CPT1a-Dependent Fatty Acid β-Oxidation
Source: Oxid Med Cell Longev. 2020 Mar 20;2020:5849794. doi: 10.1155/2020/5849794 (PMC7109578; doi:10.1155/2020/5849794)
Supplement: Supplementary Materials — Figure S1: the effects of Etomoxir and Leptin on CPT1a expression. (A, B) Representative blots of CPT1a after the use of Etomoxir and Leptin with different dose and time. (C, D) Protein expression levels of CPT1a after the use of Etomoxir and Leptin with different dose and time. The gray values were calculated, and protein expression levels were normalized to β-actin. n = 3 per group; data are expressed as mean ± SD; ∗P < 0.05 versus N group. Figure S2: the effects of mild hypothermia on glycolysis and TCA cycle. (A) Representative blots of PFKM, the key enzyme of glycolysis. (B) Representative blots of CS and IDH2, the key enzymes of TCA cycle. (C) Protein expression level of PFKM. (D) Protein expression levels of CS and IDH2. The gray values were calculated, and protein expression levels were normalized to GAPDH. n = 5 per group; data are expressed as mean ± SD; ∗P < 0.05 versus N group, #P < 0.05 versus IR group. [file 5849794.f1.docx]

**supplementary materials**

**
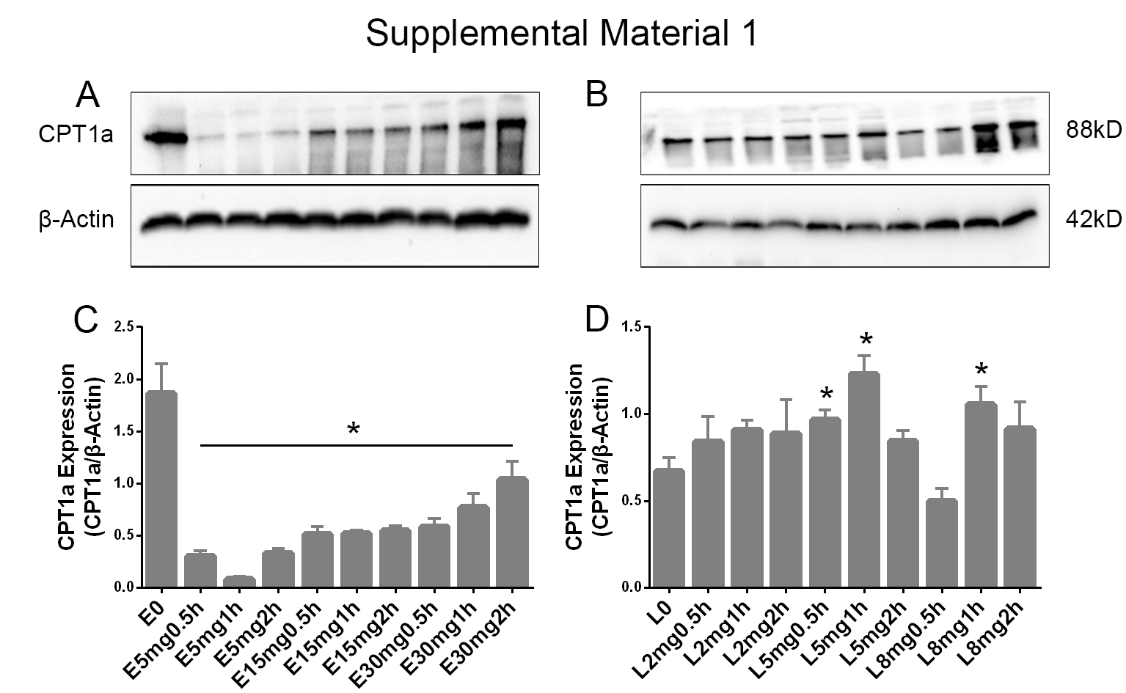
**

Figure S1. The effects of Etomoxir and Leptin on CPT1a expression. (A-B) Representative blots of CPT1a after the use of Etomoxir and Leptin with different dose and time. (C-D) Protein expression levels of CPT1a after the use of Etomoxir and Leptin with different dose and time. The gray values were calculated, and protein expression levels were normalized to β-actin. *n* = 3 per group; data are expressed as mean ± SD; ^*^ *P* < 0.05 versus N group.


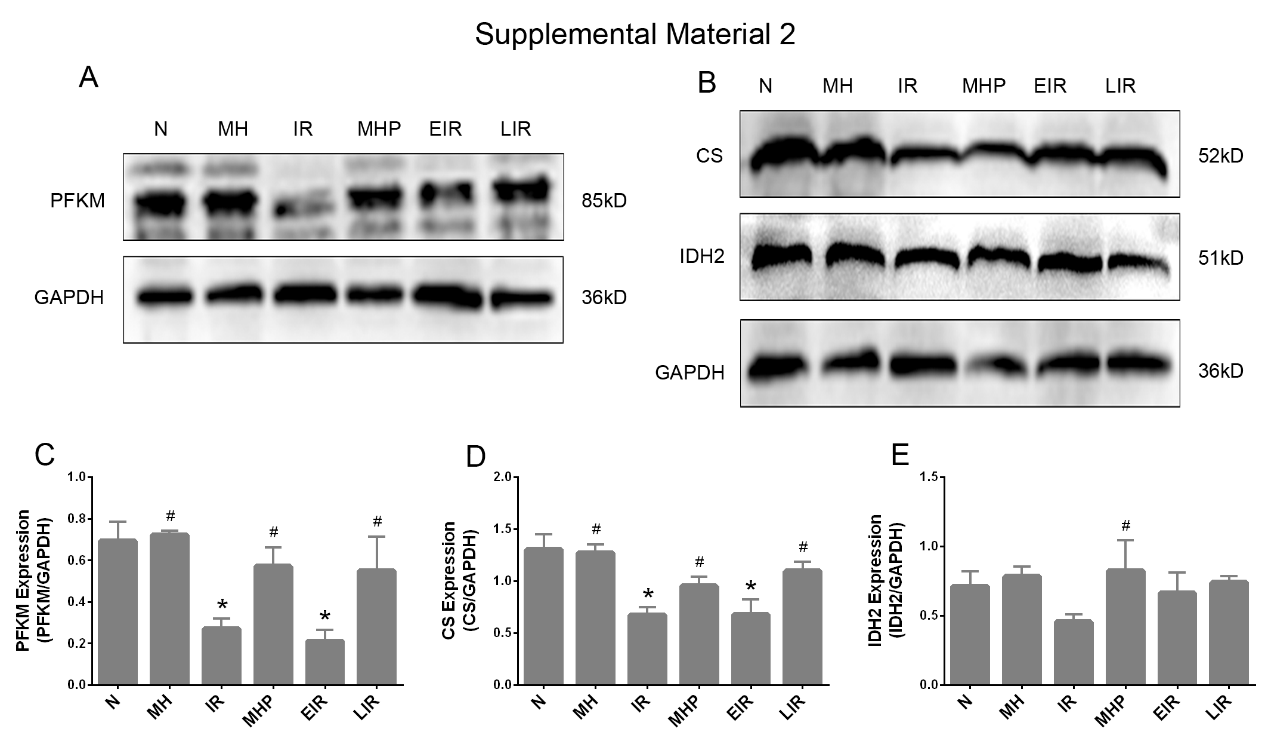


Figure S2. The effects of mild hypothermia on glycolysis and TCA cycle. (A) Representative blots of PFKM, the key enzyme of glycolysis. (B) Representative blots of CS and IDH2, the key enzymes of TCA cycle. (C) Protein expression level of PFKM. (D) Protein expression levels of CS and IDH2. The gray values were calculated, and protein expression levels were normalized to GAPDH. *n* = 5 per group; data are expressed as mean ± SD; ^*^ *P* < 0.05 versus N group, ^#^ *P* < 0.05 versus IR group.
